# A Single-Center Retrospective Analysis of Local and Distant Relapse of Breast Cancer Following Immediate Breast Reconstruction According to Molecular Subtypes

**DOI:** 10.3389/fonc.2022.912163

**Published:** 2022-06-02

**Authors:** Chunyong Han, Xuehui Zhang, Jingyan Sun, Jing Liu, Shanshan He, Jian Yin

**Affiliations:** ^1^ Department of Breast Reconstruction, The Sino-Russian Joint Research Center for Oncoplastic Breast Surgery, Tianjin Medical University Cancer Institute and Hospital, Tianjin, China; ^2^ National Clinical Research Center for Cancer, Tianjin Medical University Cancer Institute and Hospital, Tianjin, China; ^3^ Key Laboratory of Cancer Prevention and Treatment of Tianjin, Tianjin Medical University Cancer Institute and Hospital, Tianjin, China; ^4^ Tianjin Clinical Research Center for Cancer, Tianjin Medical University Cancer Institute and Hospital, Tianjin, China; ^5^ Key Laboratory of Breast Cancer Prevention and Therapy, Ministry of Education, Tianjin Medical University Cancer Institute and Hospital, Tianjin, China

**Keywords:** breast cancer, molecular subtypes, immediate breast reconstruction, locoregional recurrence, distant metastasis

## Abstract

**Purpose:**

Concerns have been raised about the oncologic safety of immediate breast reconstruction (IBR) following mastectomy for breast cancer. This study aimed to evaluate locoregional recurrence (LRR) and distant metastasis (DM) of breast cancer according to its molecular subtype in patients who underwent mastectomy alone or IBR after mastectomy.

**Methods:**

In this retrospective cohort study, consecutive breast cancer patients treated by the single senior surgeon (XZ) between February 2010 and December 2014 were eligible. In total, 389 consecutive patients were included; 295 patients underwent mastectomy alone and 94 patients underwent mastectomy with IBR. Data were retrospectively collected and analyzed for LRR and DM stratified by molecular subtypes.

**Results:**

With a median follow-up of 73 and 87.5 months, 1.69% of patients in the mastectomy alone group developed LRR compared to 0% in the reconstruction group (p = 0.342) and the total incidence of DMs was 11.52% in patients who received mastectomy alone and 7.44% in patients who received postmastectomy IBR (p = 0.262), respectively. The cumulative incidence of LRR was 2.1% vs. 0% for luminal A, 0% vs. 0% for luminal B, 0% vs. 0% for human epidermal growth factor receptor 2 (HER2)-enriched, and 4.5% vs. 0% for triple-negative in the mastectomy alone group compared to the postmastectomy IBR group. The cumulative incidence of DM was 15.5% vs. 5.7% for luminal A, 10% vs. 8.7% for luminal B, 17.3% vs. 0% for HER2-enriched, and 6.8% vs. 7.1% for triple-negative in the mastectomy alone group compared to the postmastectomy IBR group. On multivariable Cox regression analysis, lymph node metastasis was associated with an increased risk of DM in the mastectomy alone group (p = 0.03) and neoadjuvant chemotherapy was associated with an increased risk of DM in the postmastectomy IBR group (p = 0.021).

**Conclusion:**

This study suggests that IBR does not have a negative impact on the LRR and DM of breast cancer according to molecular subtypes.

## Introduction

Breast cancer is a major public health burden worldwide and remains the most common cancer in the female population. In 2020, breast cancer is the most commonly diagnosed cancer in women (24.5% of total new diagnosed cancer cases) and the leading cause of cancer death in women (15.5% of the total cancer deaths) (1). In 2021, it is estimated that breast cancer alone accounts for 30% of all new diagnoses of female cancers and 15% of all the new cancer-specific deaths (2). The incidence and mortality of breast cancer have increased over the past few decades in China (3). As the overall survival increased in the past decades, immediate breast reconstruction (IBR) had become an essential component in the comprehensive management of breast cancer. IBR after mastectomy can restore body image and improve the quality of life in breast cancer patients (4). However, there are concerns raised about the oncologic safety of IBR following mastectomy for breast cancer (5, 6). Studies reported that breast reconstruction, whether IBR or delayed breast reconstruction, in comparison with mastectomy alone, did not adversely affect locoregional recurrence (LRR) and distant metastasis (DM), which demonstrated that breast reconstruction was oncologically safe (7, 8).

Breast cancer is a heterogeneous disease with different molecular alterations. In clinical practice, breast cancer can be subdivided into four major subtypes: luminal A, luminal B, human epidermal growth factor receptor 2 (HER2)-enriched, and triple-negative (9). The different molecular subtypes lead to a difference in survival outcome of breast cancer patients and different prognoses and treatment paradigms (10, 11). Although most studies of oncological safety between patients who received mastectomy alone and breast reconstruction reported similar outcomes in LRR, DM, and overall survival (7, 8), few studies have examined the differences in oncological safety based on breast cancer molecular subtypes (12). In this study, we aim to describe LRR and DM in patients who received mastectomy alone or postmastectomy IBR by the single senior surgeon (XZ) according to breast cancer subtypes in the cohort of consecutive patients between February 2010 and December 2014.

## Methods

### Study Population

We retrospectively reviewed a consecutive cohort of 389 patients who underwent mastectomy alone and postmastectomy IBR by the single senior breast surgeon (XZ) for primary invasive breast cancer at the Tianjin Medical University Cancer Institute and Hospital between February 2010 and December 2014. We excluded patients with bilateral breast cancers, with previous disease history of invasive tumors. All of the demographic, oncologic, and reconstructive data were obtained and gathered from patients’ medical records. The 389 patients were divided into 2 groups by the type of surgical procedure: 1) patients with mastectomy alone (n = 295) and 2) patients with IBR after mastectomy (n = 94). The institutional review board of Tianjin Medical University Cancer Institute and Hospital approved this retrospective analysis.

Breast cancers were staged by two independent breast surgical oncologists according to the American Joint Committee on Cancer (AJCC) classification system, 8th edition. According to the immunohistochemical (IHC) profile of biomarkers of estrogen receptor (ER), progesterone receptor (PR), HER2, and Ki-67 (Ki-67 staining became routine IHC testing at Tianjin Medical University Cancer Institute and Hospital for breast cancer since 2009), breast cancer molecular subtypes were categorized as follows (**Supplementary Table S1**): luminal A (ER-positive or PR-positive and Ki-67 ≤14%), luminal B (ER-positive or PR-positive and Ki-67 >14%), HER2-enriched (HER2-positive), and triple-negative (ER-negative, PR-negative, and HER2-negative). HER-2 IHC test scoring 3+ and fluorescence *in situ* hybridization (FISH)-amplified breast cancer were considered HER2-positive. All HER2 IHC test scoring 2+ breast cancers were further tested for gene amplification by FISH.

### Statistical Analysis

All statistical analyses were performed using IBM SPSS Version 22.0 (IBM Corp., Armonk, NY, USA). Continuous variables were presented as the mean ± standard deviation (SD). Categorical variables were displayed by frequencies. Comparison of the demographic characteristics and recurrence or metastasis rates across the two groups was performed using Student’s t-test, chi-square analysis, or Fisher exact test where appropriate. The Kaplan–Meier method with the log-rank test was used for survival curves, and Cox proportional hazards model was carried out for multivariable analysis. All statistical tests were two-sided, and a p value of <0.05 was considered statistically significant.

Locoregional recurrence (LRR) was defined as local relapse on the chest wall and breast skin or regional recurrence of internal mammary, supraclavicular, and ipsilateral axillary nodes. Locoregional recurrence-free survival (LRRFS) was defined as the time from the date of operation to the date of LRR. Recurrence at all other sites was classified as DMs. Distant metastasis-free survival (DMFS) was defined as the time from the date of operation to the date of DM.

## Results

### The Patient Cohort

In total, 389 consecutive patients who underwent mastectomy for primary invasive breast cancer from February 2010 to December 2014 were evaluated. There were 295 patients who underwent mastectomy alone (Mx), and 94 patients underwent postmastectomy IBR (Mx+IBR). Younger age, smaller size of tumor, lower stage of cancer, and lower ratio of neoadjuvant chemotherapy and adjuvant radiotherapy were, as expected, more common among the Mx+IBR group (**Table 1**).

**Table 1:** Patient demographics, clinical and pathological characteristics, and systemic treatments in mastectomy only (Mx alone) group and mastectomy and immediate breast reconstruction (Mx+IBR) group.

	Mx alone, n = 295 Patients	Mx+IBR, n = 94 Patients,	p
Age at cancer (n, %)			<0.001
≤35	19 (6.4)	26 (27.7)	
>35, ≤50	94 (31.9)	60 (63.8)	
>50, ≤65	148 (50.2)	8 (8.5)	
>65	34 (11.5)	0 (0)	
Median/mean follow-up (months)	73/65.1	87.5/76.5	0.015
T stage (n, %)			<0.001
Tis	7 (2.4)	6 (6.4)	
1	94 (31.9)	38 (40.4)	
2	152 (51.5)	29 (30.9)	
3	26 (8.8)	2 (2.1)	
4	8 (2.7)	0 (0)	
Unknown	8 (2.7)	19 (20.2)	
Lymph node status (n, %)			<0.001
N0	156 (52.9)	46 (48.9)	
N1	85 (28.8)	20 (21.3)	
N2	24 (8.1)	5 (5.3)	
N3	29 (9.8)	5 (5.3)	
Unknown	1 (2.3)	18 (19.1)	
AJCC stage (n, %)			<0.001
0	7 (2.4)	6 (6.4)	
I	66 (22.4)	26 (27.7)	
II	157 (53.2)	36 (38.3)	
III	63 (21.4)	9 (9.6)	
IV	1 (0.3)	0 (0.0)	
Unknown	1 (0.3)	17 (18.1)	
Adjuvant radiotherapy (n, %)			0.002
Yes	85 (28.8)	12 (12.8)	
No	210 (71.2)	82 (87.2)	
Neoadjuvant chemotherapy (n, %)			0.001
Yes	47 (15.9)	3 (3.2)	
No	248 (84.1)	91 (96.8)	
Molecular subtype (n, %)			0.022
Luminal A	97 (32.9)	35 (37.2)	
Luminal B	50 (16.9)	23 (24.5)	
HER2-enriched	52 (17.6)	4 (4.3)	
TNBC	44 (14.9)	14 (14.9)	
Unknown	52 (17.6)	18 (19.1)	

The details of breast cancer subtypes in the Mx alone and Mx+IBR groups were as follows: luminal A, 97 (32.9%) vs. 35 (37.2%); luminal B, 50 (16.9%) vs. 23 (24.5%); HER2-enriched, 52 (17.6%) vs. 4 (4.3%); and triple-negative breast cancer (TNBC), 44 (14.9%) vs. 14 (14.9%). Luminal A/luminal B subtype patients had a higher rate of postmastectomy IBR. HER2-enriched subtype patients had the lowest rate of immediate reconstruction. Triple-negative subtype patients had similar rates of mastectomy alone and postmastectomy IBR (**Table 2**). **Table 2** summarizes the type of IBR and mastectomy according to molecular subtype. There was no significant difference in the type of IBR and breast skin-preserving status in the two groups.

**Table 2:** Reconstruction type and NSM status in Mx+IBR group patients.

	Luminal A, 35 patients, (n, %)	Luminal B, 23 patients, (n, %)	HER2 enriched, 4 patients, (n, %)	TNBC, 14 patients, (n, %)	Unknown, 18 patients (n, %)	p
IBR type						0.427
DTI or TE	2 (5.7)	2 (8.7)	1 (25.0)	2 (14.3)	0 (0.0)	
LD	4 (11.4)	3 (13.0)	1 (25.0)	1 (7.1)	1 (5.6)	
LD+Implant	13 (37.1)	9 (39.1)	0 (0.0)	3 (21.4)	4 (22.2)	
TRAM flap	16 (45.7)	9 (39.1)	2 (50.0)	7 (50.0)	13 (72.2)	
DIEP flap	0 (0.0)	0 (0.0)	0 (0)	1 (7.1)	0 (0.0)	
NSM status						0.332
Yes	5 (14.3)	8 (34.8)	1 (25.0)	3 (21.4)	2 (11.1)	
No	30 (85.7)	15 (65.2)	3 (75.0)	11 (78.6)	16 (88.9)	

IBR, immediate breast reconstruction; DTI, direct to implant; TE, tissue expander; LD, latissimus dorsi muscle flap; TRAM, transverse rectus abdominis myocutaneous flap; DIEP, deep inferior epigastric perforator flap; NSM, nipple-sparing mastectomy.

### The Local and Distant Relapse According to American Joint Committee on Cancer (AJCC) Staging and Subtype

**Tables 3**, **4** summarize the incidence of LRR, DM, and death. The cumulative incidence of LRR was 1.69% in patients without reconstruction compared with 0% in those with breast reconstruction (p = 0.229). In addition, the cumulative incidence of DM was 11.52% in patients with Mx alone compared with 7.44% in those with Mx+IBR (p = 0.262). During the follow-up period, the incidence of death was 3.05% in patients with Mx alone compared with 2.12% in those with Mx+IBR (p = 0.628). Remarkably, almost all of the deaths occurred in patients with stage II~III and luminal A and luminal B. Taken together, there was no significant difference in the incidence of LRR, DM, and death according to AJCC breast cancer staging and molecular subtypes.

**Table 3:** Locoregional recurrence and distant metastasis by AJCC staging in the cohort of patients.

	Mx alone, n = 295 Patients (n, %)	Mx+IBR, n = 94 Patients (n, %)	p
Overall recurrence (LRR and/or DM)	35 (11.86)	7 (7.44)	0.229
LRR	5 (1.69)	0 (0.0)	0.342
AJCC Staging			
0	0 (0.0)	0 (0.0)	NA
I	1 (1.51)	0 (0.0)	1.0
II	2 (1.27)	0 (0.0)	1.0
III	2 (3.17)	0 (0.0)	1.0
Months to LRR	33.8 ± 28.2	NA	NA
AJCC Staging			
0	NA	NA	NA
I	67	NA	NA
II	43 ± 26.8	NA	NA
III	8 ± 1.41	NA	NA
DM	34 (11.52)	7 (7.44)	0.262
AJCC Staging			
0	0 (0)	0 (0)	NA
I	4 (6.06)	2 (7.69)	1.0
II	14 (8.91)	2 (5.55)	0.741
III	16 (25.39)	3 (33.34)	0.690
Months to DM	34.85 ± 25.2	65 ± 32.6	0.009
AJCC Staging			
0	NA	NA	NA
I	48.25 ± 36.17	88.5 ± 14.84	0.222
II	35 ± 25.97	88 ± 18.38	0.016
III	31.37 ± 22.33	34 ± 19.67	0.852
Death	9 (3.05)	2 (2.12)	0.628
AJCC Staging			
0	0	0	NA
I	0	0	NA
II	4 (2.54)	1 (2.77)	0.938
III	5 (7.94)	1 (11.11)	0.756

LRR, locoregional recurrence; DM, distant metastasis; NA, not applicable.

**Table 4:** Locoregional recurrence and/or distant metastasis of breast cancer by molecular subtype.

Subtype	Mx alone, n = 295 Patients, (n, %)				Mx+IBR, n = 94 Patients, (n, %)				*p	**p	***p
	N	LRR	DM	Death	N	LRR	DM	Death			
Luminal A	97	2 (2.1)	15 (15.5)	3 (3.1)	35	0 (0)	2 (5.7)	1 (2.9)	1.0	0.24	0.94
Luminal B	50	0 (0)	5 (10)	3 (6.0)	23	0 (0)	2 (8.7)	1 (4.3)	NA	1.0	0.77
HER2-enriched	52	0 (0)	9 (17.3)	1 (1.9)	4	0 (0)	0 (0)	0 (0)	NA	1.0	0.69
TNBC	44	2 (4.5)	3 (6.8)	0 (0)	14	0 (0)	1 (7.1)	0 (0)	1.0	1.0	NA
Unknown	52	1 (1.9)	2 (3.8)	2 (3.8)	18	0 (0)	2 (11.1)	0 (0)	1.0	0.27	0.27

LRR, locoregional recurrence; DM, distant metastasis; N, number; *, comparison on LRR; **, comparison on DM; ***, comparison on Death. NA, not applicable.

In **Table 5**, multivariable analysis for DM in both surgical groups was performed using the Cox proportional hazards models. Axillary lymph node metastasis significantly increased the risk of DM (p = 0.03) in the Mx alone group. Neoadjuvant chemotherapy apparently increased the risk of LRR (p = 0.031) in the Mx+IBR group.

**Table 5:** Multivariate Cox regression analysis of distant metastasis after breast surgery.

	Mx alone, n = 295 Patients			Mx+IBR, n = 94 Patients		
	HR	95% CI	P	HR	95% CI	P
Age (years)						
≤35	1.0			1.0		
>35, ≤50	0.53	0.14–1.9	0.33	0.56	0.08–3.87	0.55
>50, ≤65	0.8	0.25–2.56	0.71	2.27	0.13–38.6	0.57
>65	0.82	0.16–4.3	0.82	NA	NA	NA
Molecular subtype						
Luminal A	1.0			1.0		
Luminal B	0.55	0.19–1.59	0.27	0.82	0.07–9.27	0.87
HER2 enriched	0.93	0.36–2.34	0.87	1.0	NA	NA
TNBC	0.53	0.14–1.94	0.34	0.7	0.05–11.02	0.80
Lymph node metastasis						
No	1.0			1.0		
Yes	2.78	1.08–7.19	0.03	0.78	0.07–9.18	0.84
Neoadjuvant chemotherapy (n)						
No	1.0			1.0		
Yes	0.96	0.38–2.41	0.93	71.13	1.9–2668	0.021
Adjuvant radiotherapy (n)						
No	1.0			1.0		
Yes	1.78	0.71–4.47	0.22	4.26	0.26–70.8	0.31

NA, not applicable.

### Survival Outcome

**Figure 1:**
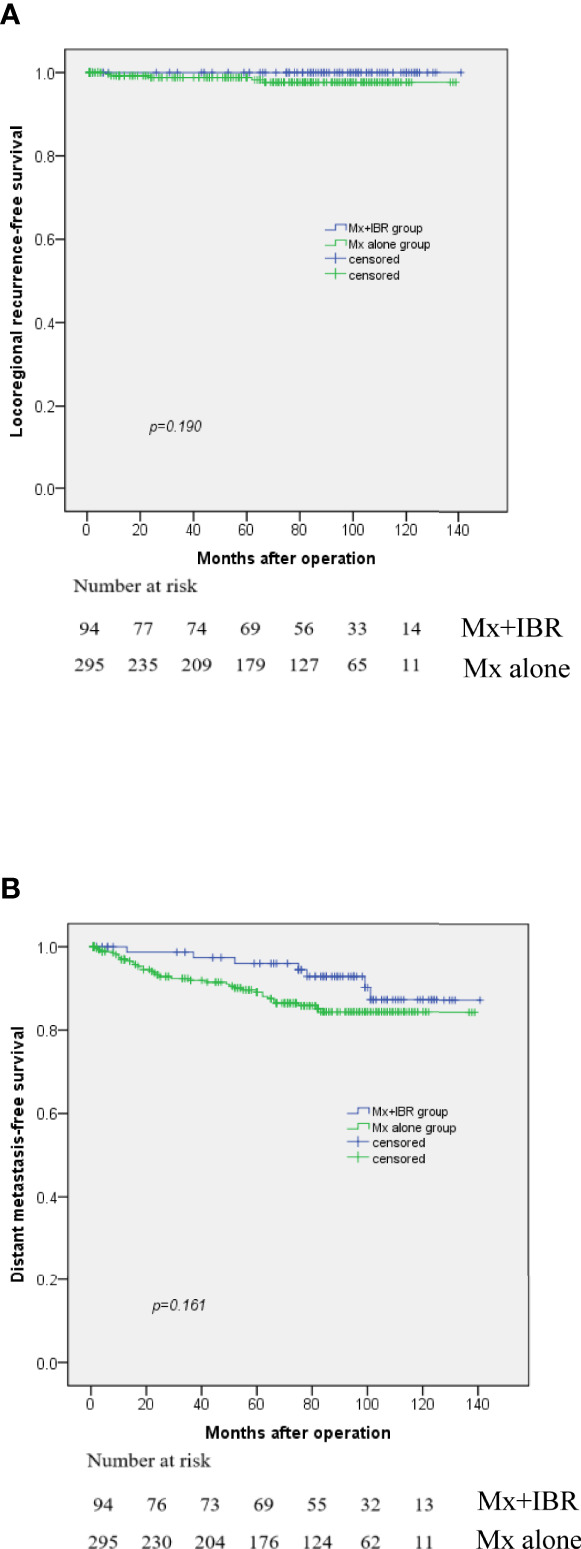
Kaplan–Meier curve for locoregional recurrence **(A)** and distant metastasis **(B)** among patients with or without immediate breast reconstruction.

As shown in **Table 1**, median follow-up was 73 months (range, 1~139 months) for the Mx alone group vs. 87.5 months (range, 1~141 months) for the Mx+IBR group. **Figure 1** reported that there was no significant difference in LRRFS and DMFS between the two groups. The overall 5-year LRRFS rates were 97.5% in the Mx alone group and 100% in the Mx+IBR group (p = 0.19). The overall 5-year DMFS rates were 85.7% in the Mx alone group and 93.1% in the Mx+IBR group (p = 0.161).

## Discussion

This study presented herein mainly focuses on the oncologic safety of postmastectomy IBR. In the cohort of 389 consecutive breast cancer patients who underwent mastectomy alone and mastectomy followed by IBR by the single senior breast surgeon (XZ) and were followed up for a median time of over 6 years, the authors failed to find any evidence for a statistically significant difference in the incidence of LRR and DM among patients who underwent mastectomy only compared to patients who underwent mastectomy followed by IBR. Moreover, the median follow-up time in the Mx+IBR group was significantly longer than that of the Mx alone group. Even during the longer follow-up period, the incidence of LRR and DM in the Mx+IBR group appears to be a little lower than that in the Mx group, though there is no significant difference. The rate of LRR in the two cohorts of our study was too low to perform any analysis and make any further significant conclusions based on molecular subtypes. Taken together, these findings argued against the notion that IBR may compromise oncologic safety of surgical management of breast cancer.

Studies reported that significant benefits in the quality of life and psychosocial well-being were gained *via* IBR after mastectomy. Breast reconstruction is increasingly provided as the essential part of postmastectomy rehabilitation (13–15). The oncologic safety of mastectomy followed by IBR is sufficiently confirmed in patients with early-stage breast cancer (7, 16–18). Our results conformed to those outcomes of other studies demonstrating a similar or higher LRRFS (16), DMFS (7, 8), and overall survival (17, 18) in the reconstruction group compared to the mastectomy alone group. In our study, patients undergoing Mx+IBR were younger, had a smaller size of tumor, and had lower cancer staging than those who underwent Mx, further confirmed by the lower rate of adjuvant radiotherapy in the Mx+IBR group; thus, baseline health and oncological status would also potentially affect the associations between disease-free survival or overall survival and the type of surgery.

In our study, patients with TNBC experienced a higher LRR than those of other subtypes in the Mx alone group, which basically conformed to previous reports, whereas patients in the Mx+IBR group had no locoregional disease relapse. Patients with HER2-enriched and luminal A in the Mx alone group and patients with luminal B in the Mx+IBR group experienced higher rates of DM than those of other subtypes. Recent reports (12, 19, 20) demonstrated higher rates of LRR and DM associated with specific molecular subtypes, which are not totally in agreement with those in our study. Holleczek et al. (19) reported the results from a population-based registry study that included 9,359 female patients with primary invasive breast cancer who underwent breast-conserving therapy or mastectomy and observed a higher cumulative incidence of LRR and DM for HER2-positive and TNBC subtypes. In another large population-based analysis that included 12,053 breast cancer patients, Ignatov et al. (20) also showed that triple-negative and HER2-enriched subtypes were associated with an increased risk of LRR and DM. The disagreement between our study and the published reports could be explained to some extent according to oncological characteristics. In our cohort of Mx group, the luminal A and HER2 breast cancer patients who experienced DM during follow-up were mostly staged into N2~N3. There are 9 (60%) and 5 (55.5%) patients staged into N2~N3 in 15 luminal A and nine HER2-enriched metastatic patients, respectively, which were markedly higher than the luminal B subtype (1 patient, 20%) and TNBC subtype (no N2 or N3 patients). Most of these N2~N3 breast cancer patients did not receive neoadjuvant chemotherapy. Moreover, due to economic problems, most HER2-enriched breast cancer patients in China failed to receive HER2-targeted treatment (trastuzumab) because Herceptin was not covered by Chinese medical insurance during 2010 to 2014. Remarkably, in the Mx group, 80% (4 in 5) of the patients who developed an LRR were also found to develop DMs. This finding confirms the late-stage baseline oncological status of this metastatic subgroup and the need for making a strengthening treatment plan in these patients suffering from locoregional relapse. In the Mx+IBR group, 4 out of 7 (57.1%) patients were staged into N2~N3, which further indicated that late-stage breast cancer patients, especially with N2~N3 lymph node metastasis, were recommended to receive the delayed breast reconstruction rather than IBR after mastectomy.

Postmastectomy IBR has become a common and widely used procedure. To reconstruct the superior cosmetic breasts, skin-sparing mastectomy (SSM) and nipple-sparing mastectomy (NSM) are preferred when the surgeons perform the IBR. Immediate implant and autologous breast reconstruction can be safely performed with a similar or higher survival outcome compared to mastectomy only patients (18). Due to the safety of IBR and the advance of materials and surgical technique, some novel breast reconstruction options are invented and performed, such as pre-pectoral implant breast reconstruction (21). As the field of breast reconstruction continues to evolve, other modalities aimed to optimize the esthetic reconstructed breasts are on the horizon and will become a reality in the near future.

However, there are several limitations in our study: first, it is a consecutive retrospective study by the single senior surgeon in our hospital. Second, molecular classification is based on IHC staining of ER, PR, HER2, and Ki-67; however, over 95% unknown molecular subtypes were a result of inaccessible FISH testing for HER2 IHC 2+ patients. Also, most HER2-positive patients did not receive trastuzumab treatment due to economic problems. Third, in some specific subtypes, especially luminal A, there was a higher proportion of N2~N3 lymph node metastatic patients. Moreover, due to the relatively small number of patients, further propensity score matching-based comparison analysis failed to be carried out, which may lead to a potential bias.

## Conclusion

Our results suggest no oncological unsafety in breast cancer patients who underwent mastectomy alone and IBR after mastectomy. We further performed the comparison analysis on LRR and DMs between two groups stratified by breast cancer molecular subtypes. We did not identify molecular subtypes as a risk factor of LRR and DMs, which further confirmed the oncological safety of IBR. During the limitation of this study, additional studies will be required to further confirm the oncological safety of IBR in each subtype. Further investigations into breast cancer relapse (local recurrence and DM) biomarkers may profoundly affect the indications and the timing for performing breast reconstruction.

## Data Availability Statement

The raw data supporting the conclusions of this article will be made available by the authors without undue reservation.

## Ethics Statement

The studies involving human participants were reviewed and approved by the institutional review board of Tianjin Medical University Cancer Institute and Hospital. The patients/participants provided their written informed consent to participate in this study.

## Author Contributions

CH and JY carried out the study conception and design. CH performed the data analysis and study interpretation and drafted the article. JY reviewed and revised the article. XZ performed all of the surgeries. JS, JL, and SH participated in data collection. All authors read and approved the final article.

## Funding

This work was supported by the National Natural Science Foundation of China (Nos. 82072942, 82073138, 81602341, and 81702637); Tianjin Medical University Cancer Institute and Hospital Innovative and Excellent Young Talents Program (2018-1-40); and Tianjin “The Belt and Road” Technological Innovation and Cooperation Grant (No. 18PTZWHZ00050).

## Conflict of Interest

The authors declare that the research was conducted in the absence of any commercial or financial relationships that could be construed as a potential conflict of interest.

## Publisher’s Note

All claims expressed in this article are solely those of the authors and do not necessarily represent those of their affiliated organizations, or those of the publisher, the editors and the reviewers. Any product that may be evaluated in this article, or claim that may be made by its manufacturer, is not guaranteed or endorsed by the publisher.

## Abbreviations

TNBC, triple-negative breast cancer; ER, estrogen receptor; PR, progesterone receptor; HER2, human epidermal growth factor receptor 2; IHC, immunohistochemical; FISH, fluorescence *in situ* hybridization; Mx, mastectomy; IBR+Mx, postmastectomy immediate breast reconstruction; LRR, locoregional recurrence; DM, distant metastasis; LRRFS, locoregional recurrence-free survival.
