# Supplementary material for: A Single-Center Retrospective Analysis of Local and Distant Relapse of Breast Cancer Following Immediate Breast Reconstruction According to Molecular Subtypes
Source: Front Oncol. 2022 Jun 2;12:912163. doi: 10.3389/fonc.2022.912163 (PMC9201333; doi:10.3389/fonc.2022.912163)
Supplement: Supplementary file 1 [file Table_1.docx]

**Supplementary Table 1.** Definitions of breast cancer subtypes

| **Subtype** | **Definition** |
| --- | --- |
| Luminal A | HR+, HER2-, ki67=<14% |
| Luminal B | HR+, HER2-, ki67>14% |
| HER2 enriched | HR+ or HR-, HER2+ |
| TNBC | HR-, HER2- |
| Unknown | HR+ or HR-, HER2 IHC 2+, FISH unknown or not testing. |

TNBC, triple negative breast cancer; HR, hormone receptor; +, positive; -, negative; IHC, immunohistochemical testing.
